# Sex Steroid Hormone Levels and Reproductive Development of Eight-Year-Old Children following *In Utero* and Environmental Exposure to Phthalates

**DOI:** 10.1371/journal.pone.0102788

**Published:** 2014-09-10

**Authors:** Pen-Hua Su, Jia-Yuh Chen, Ching-Yi Lin, Hsiao-Yen Chen, Pao-Chi Liao, Tsung-Ho Ying, Shu-Li Wang

**Affiliations:** 1 Department of Pediatrics, Chung Shan Medical University Hospital, Taichung City, Taiwan; 2 School of Medicine, Chung Shan Medical University, Taichung City, Taiwan; 3 Division of Environmental Health and Occupational Medicine, National Health Research Institutes, Zhunan, Taiwan; 4 Department of Obstetrics and Gynecology, School of Medicine, Chung Shan Medical University, Taichung, Taiwan; 5 Department of Obstetrics and Gynecology, Chung Shan Medical University Hospital, Taichung, Taiwan; 6 Department of Environmental and Occupational Health, College of Medicine, National Cheng Kung University, Tainan, Taiwan; 7 Sustainable Environment Research Center, National Cheng Kung University, Tainan, Taiwan; 8 The Department of Public Health, Chung Shan Medical University, Taichung, Taiwan; Université de Montréal, Canada

## Abstract

*In utero* exposure to phthalates may adversely affect reproductive development in children due to the anti-androgenic properties of the pthalates. Accordingly, we aimed to determine the effects of *in utero* and environmental phthalate exposure on the reproductive development of eight-year-old children. We recruited 180 children in central Taiwan during November 2001 and followed them until August 2009 when all children became eight years old. Birth outcomes were collected. Bone age, hormone concentrations, and reproductive developmental stages were determined. Phthalate metabolite levels, including mono-2-ethylhexyl phthalate [MEHP], mono-n-butyl phthalate [MnBP], and mono-benzyl phthalate [MBzP], were assessed. No significant gender differences were found in *in utero* phthalate exposure. Maternal urinary levels of phthalate metabolites did not correlate significantly with birth outcomes, physical characteristics, and reproductive hormones of the eight-year-old children. Regarding the urinary phthalate metabolite levels of the eight-year-old children, MEHP correlated significantly with serum progesterone levels. MEHP levels in girls correlated significantly with serum progesterone levels. MnBP correlated significantly with serum FSH in all children. In girls, MnBP correlated with serum FSH, and MBzP correlated with serum progesterone and FSH levels. Urinary phthalate metabolite levels did not correlate with female developmental stages or the development of female reproductive organs. Phthalate metabolites did not correlate with the physical characteristics and reproductive hormones in boys. Therefore, environmental exposure to phthalates, as determined by urinary phthalate metabolite levels of eight-year-old children, may affect reproductive hormone levels in children, indicating that further studies on the environmental health effects of phthalates are warranted.

## Introduction

Phthalates are synthetic chemicals used primarily as plasticizers and chemical additives for various applications. Phthalates are released continuously from such products and are ubiquitous in the environment owing to their chemical properties (i.e., no chemical bonds formed between phthalates and plastics or additives). Consequently, human exposure to phthalates is widespread and common, particularly since phthalates can be absorbed through the skin, inhaled, ingested, or directly administered [1], [2].

After exposure, low-molecular-weight (LMW) phthalates are rapidly metabolized into their respective monoester and hydroxy-diester metabolites, and high-molecular-weight (HMW) phthalates are metabolized into monoesters and hydroxy-, oxy-, or carboxy-diester metabolites, which are then excreted via urine [2]. Recently, biomonitoring studies have demonstrated relatively high urinary levels of phthalate metabolites in the general population of Western countries [2]–[5]. Furthermore, concentrations in children, who are more sensitive to exogenous insults, were found to be even higher [1], [2], [6].

This exposure is of great concern, as phthalates have been suspected to have anti-androgenic and estrogenic effects, which may alter the reproductive development of children [7], [8]. Indeed, toxicological evidence suggests that some phthalates, including butyl benzyl phthalate (BBzP), di-n-butyl phthalate (DnBP), and di-(2-ethylhexyl) phthalate (DEHP), may alter or mimic estradiol (E2) *in vivo* and *in vitro* [8]. Animal studies indicate that gestational phthalate exposure is associated with adverse health outcomes, including disruption of endocrine and reproductive function and development [1]. Furthermore, it was suggested that most reproductive effects of phthalates are not exerted by phthalates *per se*, but rather their metabolites [9]. Recently, a study by Huang et al. [10] demonstrated that subjects with a glutathione S-transferase M1 null mutant genotype had not only higher urinary levels of total urinary mono-ethylhexyl phthalate (MEHP), a metabolite of DEHP, but also a significantly higher risk for estrogen-dependent diseases [10]. Additionally, studies on newborns have demonstrated associations between prenatal and early postnatal phthalate exposure with shorter anogenital distance, lower serum testosterone levels, and alterations in birth outcomes [11]–[13]. However, evidence regarding the effects of phthalates on human reproduction, particularly the effects of *in utero* exposure on the reproductive development of pre-pubertal children, is still very limited [14].

In the present study, we aimed to examine the association between *in utero* and environmental exposure to phthalate metabolites and the reproductive development of pre-pubertal (i.e., age 8 years) children in Taiwan. The correlations between *in utero* exposure levels and birth outcomes, reproductive hormone levels, and female sexual characteristics were analyzed.

## Methods

### Participants

The present study was a prospective follow-up study of 130 children and their mothers, who were recruited, and were followed from November 2001 to August 2009. This study of the general population in central Taiwan served as pilot for a nation-wide Taiwan Maternal and Infant Cohort Study (TMICS). All children were eight years old at the time of follow-up. Subject recruitment was previously described. This study was approved by the ethics review committee of the National Health Research Institutes in Taiwan, and parental written informed consent was obtained for each participant.

### Data and specimen collection

Participants’ characteristics at birth and age eight years were determined from records. Data on birth outcomes included sex, gestational age, birth length, birth weight, head circumference, and breast circumference. At age eight years, height, weight, body mass index (BMI), systolic blood pressure (SBP), and diastolic blood pressure (DBP) were recorded.

At follow-up, bone age (BA) was determined by examining left hand radiographs using the method of Greulich and Pyle [15], and the BA/chronological age (CA) ratio was calculated. Additionally, eight-hour fasting blood samples were obtained from each participant, immediately centrifuged, and the serum was separated and frozen at −70°C for further analysis.

### Reproductive hormone measurements

Serum total testosterone (TT) and progesterone levels were measured using a solid-phase, competitive chemiluminescent enzyme immunoassay (Immulite 2000 Systems Analyzers, Siemens Medical Solutions Diagnostics, Deerfield, Illinois). The sensitivity for TT was 1.5 ng/mL, and for progesterone was 0.1 ng/mL (0.3 nmol/L). Free testosterone levels were determined by a Coat-A-Count Free Testosterone ^125^I radioimmunoassay kit purchased from Diagnostic Products Corp. (Los Angeles, California), which had a sensitivity of 0.15 pg/mL. Serum estradiol (E2) levels were measured using a radioimmunoassay kit (Diagnostic Systems Laboratories, Santa Monica, California), and the sensitivity of this assay was 0.22 ng/dL. Serum follicle-stimulating (FSH) levels were measured via enzyme immunoassays (FSH: Abbott Laboratories, Rome, Italy; LH: Dade Behring, Milan, Italy). The sensitivity of FSH assays was 0.2 mIU/mL.

### Evaluation of reproductive development

All children underwent detailed examinations for reproductive development, which was scored by the same investigator referring to Tanner staging criteria based on development of genital, breast and pubic hair [16], [17].

### Phthalate metabolite measurements

Urinary phthalate metabolite levels were determined, as previously described [6]. Briefly, maternal urine was collected during the third trimester of pregnancy (28–36 weeks) and urine samples of children were obtained at age eight years. Urine samples were collected with a glass beaker. The samples were subsequently transferred into amber glass bottles and stored at −20°C for further analyses. Urinary concentrations of phthalate metabolites (i.e., mono-2-ethylhexyl phthalate [MEHP], mono-(2-ethyl-5-hydroxyhexyl) phthalate [5OHMEHP], mono-(2-ethyl-5-oxohexyl) phthalate [5oxo-MEHP], mono-n-butyl phthalate [MnBP], mono-benzyl phthalate [MBzP], monomethyl phthalate [MMP], and mono-ethyl phthalate [MEP]) were determined with liquid chromatography linked to tandem mass spectrometry (LC-MS/MS) by Dr. Jürgen Angerer’s laboratory at the University of Erlangen in Germany, as previously described [18], [19]. Metabolite concentrations were expressed as µg/L. Total DEHP levels indicated the sum of MEHP, 5OH-MEHP, and 5oxo-MEHP levels. Variability in urinary output was compensated for by adjusting phthalate metabolite concentrations to urinary creatinine levels. Metabolite levels that were adjusted for creatinine were expressed as micrograms per grams of creatinine (µg/gC). Urinary creatinine levels were measured at the Kaohsiung Medical University Chung-Ho Memorial Hospital using spectrophotometric methods, with picric acid as the reactive and the reader set at 520 nm.

### Statistical analysis

Birth outcomes, children’s characteristics, and sexual characteristics (Tanner scores) of girls were summarized as means and standard deviations (mean ± SD) for continuous variables and n (%) for categorical variables. Sex differences were compared by using a two-sample t-test, if data were normally distributed, or a Mann-Whitney U-test, if data were not normally distributed, for continuous variables, and a Pearson Chi-square test for categorical variables. Maternal and offspring phthalate metabolites with adjustments for creatinine were summarized as geometric means and 95% confidence intervals (CI). Spearman’s correlation analyses were performed to determine the relationships between maternal and offspring phthalate metabolite levels. Simple linear and binary logistic regression analyses were also performed to determine the factors associated with phthalate metabolite levels. Moreover, prior to conducting correlation and association analyses, birth outcomes, including birth weight, birth length, and head circumference, were adjusted for gestational age and expressed as a Z-score for analysis. All statistical assessments were two-tailed and considered statistically significant if *P* <0.00625 (0.05/8). Statistical analyses were performed using SPSS 15.0 statistics software (SPSS Inc, Chicago, Illinois).

## Results

### Participants’ characteristics

A total of 130 children (61 boys and 69 girls) aged eight years, were enrolled into this prospective follow-up study. The birth outcomes, specifically birth weights, birth lengths, and head circumferences of newborns stratified by gestational age are shown in Table S1. Of the 130 children included in this study, only three had a gestational age of <37 weeks (Table S1). Table 1 presents the birth outcomes, and children’s physical characteristics and reproductive hormone levels at eight years of age of the entire cohort, as well as stratified by sex. At age eight years, girls had a higher BA and BA/CA ratio than boys (*P*<0.001). Prior to conducting correlation and regression analyses, the Z-scores of all birth outcomes for gestational age were calculated (Table S2). For the sexual characteristics of eight-year-old children, we did not observe any boy who had enter the Tanner stage 2. Of the 69 girls, 42 (60.9%) were classified at Tanner stage 1, 22 (31.9%) at Tanner stage 2 and 5 (7.3%) at Tanner stage 3 (Table 1).

**Table 1:** Birth outcomes, and children’s physical characteristics, reproductive hormone levels and sexual characteristics at eight years of age.

Variables	n	Total	Boys	Girls	*P*-value
		(n = 130)	(n = 61)	(n = 69)	
***Birth outcomes***					
Gestational age, weeks	86	38.71 ± 2.16	38.86 ± 1.27	38.55 ± 2.82	0.746
Birth length, cm	121	51.43 ± 2.29	51.82 ± 1.97	51.11 ± 2.50	0.172
Birth weight, g	121	3136.84 ± 405.15	3217.09 ±368.52	3069.97 ± 424.52	0.046
Head circumference, cm	121	33.58 ± 1.31	33.65 ± 1.20	33.52 ± 1.41	0.535
Breast circumference, cm	121	32.92 ± 1.59	32.95 ± 1.50	32.89 ± 1.68	0.877
Preterm	86	14 (16.3%)	7(15.9%)	7 (16.7%)	1
***Children at 8 years of age***					
Height, cm	130	130.69 ± 5.41	130.57 ± 5.97	130.8 ± 4.91	0.968
Weight, kg	130	29.18 ± 7.1	29.36 ± 6.07	29.02 ± 7.93	0.808
Body mass index, kg/m^2^	130	16.94 ± 3.14	17.11 ± 2.64	16.79 ± 3.53	0.155
Systolic blood pressure, mmHg	130	98.7 ± 8.87	99.25 ± 7.58	98.22 ± 9.9	0.377
Diastolic blood pressure, mmHg	130	63.16 ± 7.9	62.43 ± 6.49	63.8 ± 8.97	0.314
Bone age	130	7.36 ± 2.08	5.93 ± 1.39	8.62 ± 1.76	<0.001*
Bone age/chronological age ratio	130	0.92 ± 0.26	0.74 ± 0.16	1.08 ± 0.22	<0.001*
Estradiol, ng/dL^a^	126	2.32 ± 1.53	2.12 ± 1.51	2.5 ± 1.54	0.151
Follicle-stimulating hormone, mIU/mL^a^	125	5.37 ± 11.38	4.42 ± 10.05	6.24 ± 12.5	0.681
Free testosterone, pg/mL^a^	130	0.01 ± 0.07	0 ± 0	0.02 ± 0.09	0.328
Progesterone, ng/mL^a^	130	0.15 ± 0.17	0.14 ± 0.19	0.15 ± 0.15	0.108
Total testosterone, ng/mL^a^	123	0.26 ± 0.09	0.25 ± 0.08	0.26 ± 0.1	0.887
Tanner stage (Girls) Stage 1	69			42 (60.9%)	
Stage 2	69			22 (31.9%)	
Stage 3	69			5 (7.3%)	

Data are presented as mean±SD for continuous variables and n (%) for categorical ones.

Sex differences were compared by using a two-sample t-test, if data were normally distributed, or a Mann-Whitney U-test, if data were not normally distributed, for continuous variables, and a Pearson Chi-square test for categorical variables.

* *P*<0.00625 (0.05/8) indicates a significant difference.

a Reference values: Estradiol, 0.5–1.1 ng/dL for male, 0.5–2.0 ng/dL for female; Follicle-stimulating hormone, 0.26–3.0 mIU/mL for male, 1.0–4.2 mIU/mL for female; Free testosterone, 0.4–0.9 pg/mL for male and female; Progesterone, <0.1–0.33 ng/mL for male and female; Total testosterone, <0.03–0.1 ng/mL for male, <0.03–0.1 ng/mL for female.

(Table 710–6. In Behrman, Kliegman, Jenson, eds. Nelson Textbook of Pediatrics. Philadelphia: Saunders; 17^th^ edition, 2003:2406–2411).

### Phthalate metabolite levels of mothers and children

Table 2 presents the urinary phthalate metabolite levels (i.e., exposure levels) of mothers and their children at eight years of age. Boys had significantly higher urine creatinine levels than girls (48.7 vs. 27.71 mg/dL; *P*<0.001). After exposure levels were adjusted for creatinine, no significant differences were found between boys and girls in either *in utero* exposure levels and urinary phthalate metabolite levels at eight years of age.

**Table 2:** Phthalate metabolite levels of mothers and eight-year-old children after adjusting for creatinine.

	Maternal phthalate metabolites levels				Phthalate metabolites levels of 8-year-old children			
Phthalate metabolite parameters	Total	Boys	Girls	*P*-value	Total	Boys	Girls	*P*-value
	(n = 130)	(n = 61)	(n = 69)		(n = 130)	(n = 61)	(n = 69)	
Urine creatinine, mg/dL	59.79	65.1	55.45	0.339	36.1	48.7	27.71	<0.001*
	(52.74, 67.78)	(54.84, 77.28)	(46.15, 66.62)		(31.31, 41.63)	(39.69, 59.77)	(23.14, 33.18)	
MEHP	17.15	16.79	17.47	0.915	8.36	9.56	7.43	0.049
	(14.56, 20.19)	(13.09, 21.54)	(14.0, 21.79)		(6.74, 10.37)	(7.13, 12.81)	(5.41, 10.21)	
5OH-MEHP	7.6	9.26	6.38	0.261	43.03	45.69	40.81	0.346
	(5.49, 10.53)	(5.7, 15.04)	(4.08, 9.98)		(36.98, 50.08)	(35.96, 58.04)	(33.52, 49.7)	
5oxo-MEHP	13.29	15.06	11.9	0.579	36.6	39.68	34.07	0.165
	(10.05, 17.58)	(9.66, 23.5)	(8.31, 17.04)		(31.46, 42.58)	(31.47, 50.02)	(27.83, 41.72)	
Total DEHP	50.71	55.03	47.18	0.595	91.7	97.28	87.04	0.215
	(42.28, 60.83)	(40.75, 74.3)	(37.76, 58.95)		(78.38, 107.28)	(76.9, 123.05)	(70.19, 107.93)	
MnBP	66	64.11	67.71	0.937	93.95	84.55	103.13	0.261
	(55.65, 78.27)	(51.04, 80.53)	(52.44, 87.44)		(82.43, 107.08)	(70.22, 101.79)	(85.67, 124.15)	
MBzP	15.72	14.18	17.23	0.236	10.45	11.38	9.69	0.307
	(13.79, 17.93)	(11.61, 17.31)	(14.46, 20.52)		(8.61, 12.69)	(8.37, 15.47)	(7.54, 12.45)	
MMP	53.51	54.2	52.9	0.816	7.14	6.41	7.86	0.235
	(44.39, 64.5)	(40.73, 72.14)	(41.11, 68.06)		(5.93, 8.59)	(4.86, 8.44)	(6.09, 10.13)	
MEP	61.15	57.41	64.67	0.52	15.96	13.33	18.72	0.084
	(52.26, 71.56)	(45.46, 72.51)	(52.06, 80.34)		(13.26, 19.21)	(10.35, 17.17)	(14.32, 24.47)	

Data are presented as geometric means and 95% CI, and metabolite concentrations were expressed relative to creatinine levels (mg/g). Sex differences were compared by using the Mann-Whitney U-test.

* *P*<0.00625 (0.05/8) indicates a significant difference.

*Abbreviations:* MEHP, mono-2-ethylhexyl phthalate; 5OH-MEHP, mono-(2-ethyl-5-hydroxyhexyl) phthalate; 5oxo-MEHP, mono-(2-ethyl-5-oxohexyl) phthalate; DEHP, di-(2-ethylhexyl) phthalate; MnBP, mono-n-butyl phthalate;MBzP, mono-benzyl phthalate; MMP, monomethyl phthalate; MEP, mono-ethyl phthalate.

### Maternal urinary phthalate metabolite levels and birth outcomes

The correlations between birth outcomes and maternal urinary phthalate metabolite levels are presented in Table 3 and Table 4. No significant correlations were found between maternal urinary levels of phthalate metabolites and birth outcomes (Tables 3 and 4). Of the phthalate metabolites tested in this study, only *in utero* exposure to MMP had a negative correlation with birth weight in boys (r = −0.423, *P*  = 0.006) (Table S3). In addition, urinary phthalate metabolite levels of children age eight years did not correlate with maternal urinary levels of phthalate metabolites (Tables 3, 4, Table S3). In addition, maternal urinary levels of phthalate metabolites did not correlate with the physical characteristics and reproductive development of children aged eight years (Table S4).

**Table 3:** Effects of material urinary levels of phthalate metabolites on the birth outcome and the phthalate metabolites levels of eight-year-old children.

		MEHP			5OH-MEHP			5oxo-MEHP			Total DEHP		
Population	Outcomes	β (SE) orOR(95%CI)	*P* -value	R^2^	β (SE) orOR(95%CI)	*P* -value	R^2^	β (SE) orOR(95%CI)	*P* -value	R^2^	β (SE) orOR(95%CI)	*P* -value	R^2^
Total (n = 130)													
	***Birth outcomes***												
	Gestational age, weeks	.0040 (.0030)	0.193	0.02	.0051 (.0039)	0.201	0.019	.0027 (.0019)	0.143	0.025	.0013 (.0009)	0.157	0.024
	Birth weight, g^a^	.001 (.001)	0.355	0.011	.002 (.002)	0.169	0.023	.001 (.001)	0.209	0.019	.001 (.0004)	0.223	0.018
	Birth length, cm^a^	.002 (.001)	0.093	0.034	.002 (.002)	0.141	0.027	.001 (.001)	0.162	0.024	.001 (.0004)	0.123	0.029
	Head circumference, cm^a^	.002 (.001)	0.188	0.021	.003 (.002)	0.106	0.032	.002 (.001)	0.062	0.042	.001 (.0004)	0.09	0.035
	Sex (M vs. F)	1.027 (0.985–1.071)	0.215	0.028	1.007 (0.998–1.016)	0.133	0.033	1.006 (0.999–1.014)	0.114	0.049	1.002 (0.999–1.005)	0.199	0.029
	Phthalate metabolite levels at 8 years of age	–.0487 (.1572)	0.757	0.001	.0314 (.3778)	0.934	0	–.0364 (.1572)	0.817	0	–.0417 (.2087)	0.842	0
Boys (n = 61)													
	***Birth outcomes***												
	Gestational age, weeks	.0030 (.0018)	0.105	0.061	.0054 (.0023)	0.025	0.114	.0025 (.0011)	0.026	0.112	.0011 (.0005)	0.036	0.1
	Birth weight, g^a^	.001 (.001)	0.421	0.017	.002 (.002)	0.314	0.026	.001 (.001)	0.38	0.02	.0004 (.0004)	0.366	0.021
	Birth length, cm^a^	.002 (.001)	0.104	0.066	.003 (.002)	0.099	0.068	.001 (.001)	0.184	0.045	.001 (.0004)	0.128	0.058
	Head circumference, cm^a^	.002 (.001)	0.148	0.053	.004 (.002)	0.017	0.137	.002 (.001)	0.020*	0.132	.001 (.0004)	0.034	0.11
	Phthalate metabolite levels at 8 years of age	–.0499 (.1362)	0.715	0.002	–.0366 (.5125)	0.943	0	–.0855 (.2011)	0.672	0.003	.0917 (.2706)	0.736	0.002
Girls (n = 69)													
	***Birth outcomes***												
	Gestational age, weeks	.0224 (.0188)	0.241	0.034	–.0022 (.0184)	0.904	0	.0074 (.0145)	0.611	0.007	.0042 (.0069)	0.539	0.009
	Birth weight, g^a^	–.003 (.006)	0.664	0.005	.001 (.006)	0.878	0.001	.0004 (.005)	0.926	<.001	–.0001 (.0022)	0.956	<.001
	Birth length, cm^a^	.001 (.007)	0.891	<.001	–.006 (.006)	0.35	0.022	–.003 (.005)	0.519	0.01	–.001 (.002)	0.546	0.009
	Head circumference, cm^a^	–.002 (.007)	0.803	0.002	–.012 (.007)	0.075	0.077	–.009 (.005)	0.085	0.073	–.004 (.002)	0.117	0.06
	Phthalate metabolite levels at 8 years of age	–.0261 (.4925)	0.958	0	.1969 (.7954)	0.805	0.001	.6792 (.5953)	0.258	0.019	.4559 (.6178)	0.463	0.008

Data are presented as β (SE) and R^2^ for linear regression analysis or OR (95%.CI) and Nagelkerke R^2^for binary logistical regression analysis.

a Z-scores of birth outcomes, including body weight, body length, and head circumference, for gestational age were calculated prior to conducting correlation analysis.

* *P*<0.00625 (0.05/8) indicates a significant correlation.

*Abbreviations:* MEHP, mono-2-ethylhexyl phthalate; 5OH-MEHP, mono-(2-ethyl-5-hydroxyhexyl) phthalate; 5oxo-MEHP, mono-(2-ethyl-5-oxohexyl) phthalate; DEHP, di-(2-ethylhexyl) phthalate.

**Table 4:** Effects of material urinary levels of phthalate metabolites on the birth outcome and the phthalate metabolites level levels of eight-year-old children.

		MnBP			MBzP			MMP			MEP		
Population	Outcomes	β (SE) orOR(95%CI)	*P* -value	R^2^	β(SE) orOR(95%CI)	*P* -value	R^2^	β (SE) orOR(95%CI)	*P* -value	R^2^	β (SE) orOR(95%CI)	*P* -value	R^2^
Total (n = 130)													
	***Birth outcomes***												
	Gestational age, weeks	.0011 (.0021)	0.609	0.003	.0122 (.0149)	0.414	0.008	–.0004 (.0022)	0.861	0	.0008 (.0016)	0.607	0.003
	Birth weight, g^a^	.001 (.001)	0.367	0.01	–.006 (.007)	0.36	0.01	–.0002 (.001)	0.825	0.001	–.0001 (.001)	0.909	<.001
	Birth length, cm^a^	.001 (.001)	0.544	0.005	–.002 (.006)	0.792	0.001	–.001 (.001)	0.598	0.003	–.0001 (.001)	0.859	<.001
	Head circumference, cm^a^	.0001 (.001)	0.958	<.001	–.001 (.007)	0.85	<.001	–.001 (.001)	0.899	<.001	.0002 (.001)	0.759	0.001
	Sex (M vs. F)	0.999 (0.995–1.002)	0.337	0.01	0.972 (0.945–1.001)	0.056	0.047	1.001 (0.997–1.004)	0.741	0.001	1.000 (0.997–1.003)	0.959	0
	Phthalate metabolite levels at 8 years of age	.2298 (0.1049)	0.03	0.036	.1419 (0.3182)	0.656	0.002	–.0042 (.0192)	0.828	0	.0209 (.0460)	0.65	0.002
Boys (n = 61)													
	***Birth outcomes***												
	Gestational age, weeks	–.0006 (.0029)	0.841	0.001	.0269 (.0184)	0.151	0.048	–.0006 (.0015)	0.685	0.004	–.0010 (.0011)	0.384	0.018
	Birth weight, g^a^	.004 (.002)	0.057	0.09	–.026 (.014)	0.069	0.003	–.001 (.001)	0.265	0.032	–.001 (.001)	0.55	0.009
	Birth length, cm^a^	.005 (.002)	0.02	0.13	–.008 (.013)	0.539	0.01	–.001 (.001)	0.291	0.029	.0001 (.001)	0.918	<.001
	Head circumference, cm^a^	.002 (.002)	0.432	0.016	–.008 (.013)	0.527	0.01	–.001 (.001)	0.508	0.011	–.0002 (0.001)	0.759	0.002
	Phthalate metabolite levels at 8 years of age	.0506 (.1431)	0.725	0.002	1.4022 (1.0567)	0.19	0.029	–.0227 (.0230)	0.328	0.016	–.0345 (.0418)	0.413	0.011
Girls (n = 69)													
	***Birth outcomes***												
	Gestational age, weeks	.0019 (.0031)	0.544	0.009	.0108 (.0226)	0.635	0.006	.0001 (.0058)	0.992	0	.0045 (.0036)	0.211	0.039
	Birth weight, g^a^	.001 (.001)	0.444	0.015	.003 (.007)	0.713	0.003	.002 (.002)	0.243	0.034	.001 (.001)	0.594	0.007
	Birth length, cm^a^	.0002 (.001)	0.855	0.001	.003 (.008)	0.718	0.003	.001 (.002)	0.74	0.003	–.001 (.001)	0.578	0.008
	Head circumference, cm^a^	–.0001 (0.001)	0.952	<.001	.001 (.008)	0.811	0.001	.001 (.001)	0.540	0.009	.001 (.001)	0.407	0.017
	Phthalate metabolite levels at 8 years of age	.2997 (.1487)	0.048	0.057	–.0330 (.1827)	0.857	0	.0337 (.0339)	0.324	0.014	.1021 (.0865)	0.242	0.02

Data are presented as β (SE) and R^2^ for linear regression analysis or OR (95%.CI) and Nagelkerke R^2^for binary logistical regression analysis.

a Z-scores of birth outcomes, including body weight, body length, and head circumference, for gestational age were calculated prior to conducting correlation analysis.

* *P*<0.00625 (0.05/8) indicates a significant correlation.

*Abbreviations:* MnBP, mono-n-butyl phthalate; MBzP, mono-benzyl phthalate; MMP, monomethyl phthalate; MEP, mono-ethyl phthalate.

### Phthalate metabolites of eight-year-old children and their development

The linear correlation between the physical characteristics and reproductive development of children aged eight years and their urinary phthalate metabolite levels are presented in Table 5 and Table 6. Urinary MEHP levels correlated significantly with serum progesterone levels (β = 0.0004; SE = 0.0001; *P* = 0.006; R^2^ = 0.060) (Table 5). Additionally, urinary MEHP levels in girls correlated significantly with serum progesterone levels (β = 5.1×10^4^; SE = 1.2×10^4^; *P*<0.001; R^2^ = 0.225). Additionally, urinary levels of MnBP correlated significantly with serum FSH levels in all eight-year-old children (β = 0.0192; SE = 0.0069; *P* = 0.006; R^2^ = 0.060). In girls, MnBP levels correlated significantly with serum FSH levels (β = 0.026; SE = 0.0086; *P* = 0.004; R^2^ = 0.125). Additionally, MBzP levels in girls correlated significantly with serum FSH (β = 0.1726; SE = 0.0471; *P* = 0.001; R^2^ = 0.175) and progesterone (β = 0.0018; SE = 0.0006; P = 0.002; R^2^ = 0.137) levels (Table 6).

**Table 5:** Effects of urinary phthalate metabolite levels of eight-year-old children on their physical characteristics and reproductive development.

		MEHP			5OH-MEHP			5oxo-MEHP			Total DEHP		
Population	Outcomes	β (SE) or OR(95%CI)	*P* -value	R^2^	β(SE) or OR(95%CI)	*P* -value	R^2^	β (SE) or OR(95%CI)	*P* -value	R^2^	β (SE) or OR(95%CI)	*P* -value	R^2^
Total (n = 130)													
	Bone age	–.002 (.002)	0.223	0.012	–.001 (.001)	0.225	0.011	–.001 (.001)	0.191	0.013	–.0005 (.0004)	0.179	0.014
	Bone age/chronological age ratio	–.0002 (.0002)	0.23	0.011	–.0001 (.0001)	0.228	0.011	–.0002 (.0001)	0.197	0.013	–.6×10^−4^(.4×10^−4^)	0.183	0.014
	Estradiol, ng/dL	.0014 (.0011)	0.234	0.011	.0006 (.0005)	0.296	0.009	.0007 (.0007)	0.327	0.008	.0003 (.0003)	0.256	0.01
	Follicle-stimulating hormone, mIU/mL	.0144 (.0085)	0.091	0.023	–.0027 (.0040)	0.5	0.004	–.0032 (.0053)	0.543	0.003	–.0003 (.0020)	0.868	0
	Free testosterone, pg/mL	–.6×10^–5^ (5.1×10^–5^ )	0.907	0	.3×10^–5^ (2.4×10^–5^)	0.907	0	.5×10^–5^ (3.2×10^–5^)	0.873	0	.1 ×10^–5^ (1.2×10^–5^)	0.929	0
	Progesterone, ng/mL	**.0004 (.0001)** *	**0.006** *	**0.06**	–.0003 (.0006)	0.609	0.002	–.0004 (.0008)	0.617	0.002	–.5×10^–5^ (2.9×10^–5^)	0.851	0
	Total testosterone, ng/mL	–.0004 (.0007)	0.528	0.003	–.0005 (.0003)	0.152	0.017	–.0006 (.0004)	0.127	0.019	–.0002 (.0002)	0.162	0.016
Boys (n = 61)													
	Bone age	–.001 (.002)	0.575	0.005	.0003 (0.0006)	0.567	0.006	–.0004 (.0008)	0.563	0.006	–.0002 (.0003)	0.566	0.006
	Bone age/chronological age ratio	–1.2×10^–4^ (2.2×10^–4^)	0.586	0.005	.4×10^–4^ (.7×10^–4^)	0.562	0.006	–.5×10^–4^ (.9×10^–4^ )	0.566	0.006	–.18 ×10^–4^ (.32×10^–4^)	0.566	0.006
	Estradiol, ng/dL	.0027 (.0020)	0.187	0.03	.0008 (.0006)	0.17	0.032	.0011 (.0008)	0.17	0.032	.0004 (.0003)	0.171	0.032
	Follicle–stimulating hormone, mIU/mL	–.0081 (.0138)	0.559	0.006	–.0029 (.0042)	0.493	0.008	–.0039 (.0055)	0.48	0.009	–.0014 (.0020)	0.497	0.008
	Free testosterone, pg/mL	–.1×10^–5^ (.4×10^–5^)	0.834	0.001	–.4×10^–6^ (1.3×10^–6)^	0.765	0.002	–.5×10^–6^ (1.7×10^–6^)	0.767	0.002	–.2×10^–6^ (.6×10^–6^)	0.776	0.001
	Progesterone, ng/mL	–.4×10^–4^ (2.6×10^–4^)	0.875	0	.3×10^–6^ (79×10^–6^)	0.997	0	.1×10^–5^ (10.5×10^–5^)	0.993	0	–.7×10^–6^ (38.6×10^–6^)	0.985	0
	Total testosterone, ng/mL	–.0002 (.0001)	0.193	0.03	–.0005 (.0003)	0.133	0.04	–.0007 (.0005)	0.12	0.043	–.0003 (.0002)	0.135	0.039
Girls (n = 69)													
	Bone age	–.003 (.002)	0.087	0.043	–.001 (.001)	0.325	0.014	–.002 (.002)	0.234	0.021	–.001 (.001)	0.129	0.034
	Bone age/chronological age ratio	–3.3×10^–4^ (1.9×10^–4^)	0.087	0.043	–1.5×10^–4^ (1.5×10^–4^ )	0.329	0.014	–2.2×10^–4^ (.1.9×10^–4^)	0.239	0.021	–1.1×10^–4^ (.7×10^–4^ )	0.131	0.034
	Estradiol, ng/dL	.0007 (.0014)	0.608	0.004	–.0001 (.0011)	0.951	0	–.0002 (.0013)	0.888	0	.0001 (.0005)	0.918	0
	Follicle–stimulating hormone, mIU/mL	.0237 (.0109)	0.033	0.07	–.0015 (.0087)	0.86	0.001	–.0006 (.0108)	0.957	0	.0026 (.0040)	0.516	0.007
	Free testosterone, pg/mL	–1.1×10^–5^ (8.4×10^–5^)	0.896	0	–19.1×10^–6^ (64.8×10^–6^)	0.769	0.001	27.1×10^–6^ (80.9×10^–6^)	0.739	0.002	6.5×10^–6^ (30.2×10^–6^)	0.83	0.001
	Progesterone, ng/mL	**5.1×10^–4^ (1.2×10^–4^)**	**<.001** *	**0.225**	–113.0×10^–6^ (103×10^–6^)	0.275	0.019	–1.3×10^–5^ (12.8×10^–5^)	0.304	0.016	22.6×10^–6^ (.48.2×10^–6^)	0.641	0.003
	Total testosterone, ng/mL	.2×10^–5^ (8.7×10^–5^)	0.98	0	–.0003 (.0007)	0.684	0.003	–.0004 (.0008)	0.609	0.004	–.0001 (.0003)	0.71	0.002
	Tanner stage			0.014			0.027			0.025			0.021
	Stage 1	Reference			Reference			Reference			Reference		
	Stage 2	1.000 (0.995, 1.004)	0.822		1.002 (0.998, 1.005)	0.358		1.002 (0.998, 1.006)	0.375		1.001 (0.999, 1.002)	0.448	
	Stage 3	0.976 (0.895, 1.064)	0.578		0.995 (0.970, 1.021)	0.705		0.994 (00964, 1.024)	0.684		0.997 (0.985, 1.009)	0.631	

Data are presented as β (SE) and R^2^ for linear regression analysis or OR (95%.CI) and Nagelkerke R^2^for binary logistical regression analysis.

* *P*<0.00625 (0.05/8) indicates a significant correlation.

*Abbreviations:* MEHP, mono-2-ethylhexyl phthalate; 5OH-MEHP, mono-(2-ethyl-5-hydroxyhexyl) phthalate; 5oxo-MEHP, mono-(2-ethyl-5-oxohexyl) phthalate; DEHP, di-(2-ethylhexyl) phthalate.

**Table 6:** Effects of urinary phthalate metabolite levels of eight-year-old children on their physical characteristics and reproductive development.

		MnBP			MBzP			MMP			MEP		
Population	Outcomes	β (SE) orOR(95%CI)	*P* -value	R^2^	β(SE) orOR(95%CI)	*P* -value	R^2^	β (SE) orOR(95%CI)	*P* -value	R^2^	β (SE) orOR(95%CI)	*P* -value	R^2^
Total (n = 130)													
	Bone age	–.0012 (.0013)	0.336	0.007	–.0035 (.0032)	0.269	0.01	.0021 (.0088)	0.807	0	.0036 (.0026)	0.169	0.015
	Bone age/chronological age ratio	–.0001 (.0002)	0.369	0.006	–.0004 (.0004)	0.27	0.01	.0003 (.0011)	0.781	0.001	.0005 (.0003)	0.146	0.016
	Estradiol, ng/dL	–.0004 (.0009)	0.67	0.001	–.0018 (.0023)	0.44	0.005	–.0085 (.0064)	0.186	0.014	–.0007 (.0019)	0.713	0.001
	Follicle-stimulating hormone, mIU/mL	**.0192 (.0069)**	**0.006** *	**0.06**	.0124 (.0174)	0.478	0.004	.0135 (.0479)	0.778	0.001	–.0057 (.0144)	0.692	0.001
	Free testosterone, pg/mL	–2.0×10^–5^ (4.2×10^–5^)	0.633	0.002	–2.6×10^–5^ (10.4×10^–5^)	0.806	0	–12.9×10^–5^ (28.6×10^–5^)	0.652	0.002	9.73×10^–5^ (8.55×10^–5^)	0.257	0.01
	Progesterone, ng/mL	.0002 (.0001)	0.147	0.017	.00001 (.00026)	0.977	0	–.0007 (.0007)	0.319	0.008	–.0001 (.0002)	0.608	0.002
	Total testosterone, ng/mL	–.00003 (.00006)	0.609	0.002	–.00002 (.00014)	0.868	0	–.00045 (.00038)	0.236	0.012	.00014 (.00011)	0.235	0.012
Boys (n = 61)													
	Bone age	.0002 (.0017)	0.883	0	–.0003 (.0023)	0.902	0	.0060 (.0087)	0.498	0.008	.0005 (.0037)	0.902	0
	Bone age/chronological age ratio	.0001 (.0002)	0.784	0.001	–.00003 (.0003)	0.914	0	.0008 (.0010)	0.406	0.012	.0001 (.0004)	0.827	0.001
	Estradiol, ng/dL	–.0021 (0.0018)	0.256	0.022	–.0031 (.0025)	0.212	0.027	–.0182 (.0092)	0.053	0.063	–.0019 (.0040)	0.63	0.004
	Follicle–stimulating hormone, mIU/mL	–.0005 (.0121)	0.964	0	–.0117 (.0167)	0.487	0.008	–.0324 (.0633)	0.611	0.004	–.0176 (.0264)	0.508	0.008
	Free testosterone, pg/mL	–.2×10^–5^ (.4×10^–5^)	0.636	0.004	–.1×10^–5^ (.5×10^–5^)	0.885	0	–.7×10^–5^ (2.0×10^–5^)	0.717	0.002	–.4×10^–5^ (.8×10^–5^)	0.64	0.004
	Progesterone, ng/mL	–.0002 (.0002)	0.495	0.008	–.0003 (.0003)	0.379	0.013	–.0012 (.0012)	0.33	0.016	–.0002 (.0005)	0.642	0.004
	Total testosterone, ng/mL	–.00007 (.00010)	0.48	0.009	.00002 (.00014)	0.905	0	–.00057 (.00053)	0.282	0.021	.00013 (.00022)	0.57	0.006
Girls (n = 69)													
	Bone age	–.0034 (.0012)	0.007	0.105	–.0072 (.0072)	0.321	0.015	–.0053 (.0010)	0.597	0.004	.0017 (.0026)	0.504	0.007
	Bone age/chronological age ratio	–.0004 (.0002)	0.007	0.103	–.0009 (.0009)	0.336	0.014	–.0007 (.0012)	0.584	0.005	.0002 (.0003)	0.485	0.007
	Estradiol, ng/dL	–.00003 (.0011)	0.982	0	.0084 (.0063)	0.189	0.027	–.0013 (.0088)	0.88	0	–.0008 (.0022)	0.73	0.002
	Follicle–stimulating hormone, mIU/mL	**.0260 (.0086)**	**0.004** *	**0.125**	**.1726 (.0471)**	**0.001** *	**0.175**	.0475 (.0711)	0.506	0.048	–.0043 (.0183)	0.816	0.001
	Free Testosterone, pg/mL	–3.77×10^–5^ (6.88×10^–5^)	0.586	0.005	–8.8×10^–5^ (38.8×10^–5^)	0.82	0.001	–26.1×10^–5^ (53.3×10^–5^)	0.626	0.004	11.2×10^–5^ (13.6×10^–5^)	0.413	0.01
	Progesterone, ng/mL	.0003 (.0001)	0.014	0.091	**.0018 (.0006)**	**0.002** *	**0.137**	–.0004 (.0009)	0.676	0.003	–.0001 (.0002)	0.698	0.002
	Total testosterone, ng/mL	–.00002 (.00007)	0.827	0.001	–.00025 (.00040)	0.535	0.006	–.00036 (.00055)	0.512	0.007	.00014 (.00014)	0.331	0.015
	Tanner stage			0.028			0.017			0.039			0.023
	Stage 1	Reference			Reference			Reference			Reference		
	Stage 2	0.997 (0.992, 1.002)	0.205		1.005 (0.989, 1.022)	0.545		1.009 (0.986, 1.034)	0.436		1.002 (0.996, 1.008)	0.517	
	Stage 3	0.994 (0.979, 1.009)	0.423		0.980 (0.912, 1.053)	0.586		0.927 (0.789, 1.089)	0.354		0.987 (0.944, 1.032)	0.564	

Data are presented as β (SE) and R^2^ for linear regression analysis or OR (95%.CI) and Nagelkerke R^2^ for binary logistical regression analysis.

* *P*<0.00625 (0.05/8) indicates a significant correlation.

*Abbreviations:* MnBP, mono-n-butyl phthalate; MBzP, mono-benzyl phthalate; MMP, monomethyl phthalate; MEP, mono-ethyl phthalate.

No correlations were shown between urinary phthalate metabolite levels of boys aged eight years with physical characteristics and reproductive development. Lastly, urinary phthalate metabolite levels of eight-year-old children did not correlate with either their physical characteristics or reproductive development (Table S5).

## Discussion

Epidemiological studies have indicated that phthalates may affect reproductive outcomes in children; however, the health effects of specific phthalates in children, in particular DEHP and its metabolites, are unknown [6], [14]. In the present study, we examined birth outcomes, reproductive hormone levels, and markers of reproductive development of eight-year-old male and female children by following *in utero* and environmental exposure to phthalates. No significant differences were found in *in utero* phthalate exposure between boys and girls, indicating that both boys and girls in this study were exposed to similar levels of phthalate *in utero*.

Phthalates have been previously implicated in affecting fetal development, and in turn, birth outcomes. In a cohort of 404 infants, Wolff *et al*. [13] demonstrated that LMW phthalate metabolites, but not DEHP or HMW phthalate metabolites, were positively associated with gestational age and head circumference after adjusting for multiple factors, including creatinine levels. However, no associations were found between *in utero* phthalate levels and birth weights or birth lengths of newborns. In the present study, with the exception of one circumstance, *in utero* exposure to phthalates did not correlate with birth outcomes. Specifically, maternal urinary MMP levels were found to correlate negatively with birth weight in boys.

Phthalate metabolites have short biological half-lives and are rapidly excreted via urine [2]. This is supported by our finding that maternal phthalate levels did not correlate with those of their eight-year-old children. Therefore, we have originally comsidered that maternal/prenatal phthalate exposure and environmental phthalate exposure of 8-year-old children can be treated as two different entities that can be investigated separately. However, previous investigations have shown that prenatal phthalate exposure is associated with childhood behavior at ages 4–9 years [20], neurobehavioral development at ages 6–10 years [21], male-typical play (masculine play) behavior in boys at ages 3.6–6.4 years [22] and childhood eczema at age 2 years [23]. Therefore, as in the present study, it is also reasonable to hypothesize that prenatal phthalate exposure (maternal exposure) may affect sex steroid hormone levels and reproductive development of 8-year-old children. However, the outcomes of our study did not support the original hypothesis that maternal urinary levels of phthalate metabolites did not correlate with the physical characteristics and reproductive development of eight-year-old children (Table S4), which emphasizes the need for additional study.

Phthalates have been suspected to have anti-androgenic and estrogenic effects, which may alter the reproductive development of children [7], [8]. In fact, some phthalates have been shown to alter or mimic E2 *in vivo* and *in vitro* [8]. In terms of environmental exposure to phthalates, as determined by the urinary phthalate metabolite levels of eight-year-old children, certain phthalate metabolites were indeed found to affect their physical characteristics and reproductive hormone levels. Regarding the reproductive hormone of the entire cohort, only MEHP correlated significantly with serum progesterone levels and MnBP correlated significantly with serum FSH levels. Urinary phthalate metabolite levels did not correlate with the physical characteristics and reproductive hormone levels of eight-year-old boys. Conversely, in girls, MEHP and MBzP were found to correlate positively with serum progesterone levels, and MnBP and MBzP correlated positively with serum FSH levels. Indeed, we found that for every 1 µg/L increase in urinary MBzP levels of girls aged eight years, there was an increase in serum FSH levels of 0.1726 mIU/mL (β = 0.1726, SE = 0.0471; *P* = 0.001). While exposure to some phthalates was associated with alterations in reproductive hormone levels in girls, none of the phthalates studied affected female reproductive development. These findings corroborate the results of another study, which demonstrated that phthalate exposure is not associated with precocious puberty in female children aged roughly seven years [24].

This study has a few limitations that need to be addressed. First, in addition to using a single urine specimen to determine maternal urinary phthalate levels during the third trimester of pregnancy, we did not measure phthalate levels during early pregnancy (i.e., the first trimester), which is when sex determination and differentiation of the fetus occurs. However, Braun *et al*. (2012) had previously determined that, with the exception of BPA and MBzP, the variability of most urinary phthalate metabolites was similar before and during pregnancy, suggesting that maternal urinary phthalate levels may be measured at any stage of pregnancy to determine *in utero* exposure to phthalates [25]. Second, given that we studied pre-pubertal children, it may be too early to determine the effects of phthalate exposure on gonadal development in these children by evaluating ovary follicles, endometrial development and uterus length. However, precocious puberty is defined as the development of pubertal changes at an age younger than the accepted lower limits for age at onset of puberty, namely, before age 8 years in girls and 9 years in boys [26]. Since we aimed to investigate the association between environmental phthalate exposure and abnormal reproductive development, we did not want to miss the disease onset time of CPP and thus started to follow the subjects at 8 to 9 years. Third, it was determined that urinary phthalate metabolite levels have an effect on certain sex hormone levels (i.e., FSH and progesterone in girls). For boys, sertoli cell markers (AMH and inhibin B) in serum are reliable markers for evaluation of basal testicular function in childhood [27], but we did not measure the serum levels of AMH and inhibin B in this research project. Of note, we have been continuously following this cohort of children since birth and they are now 12 years of age. Accordingly, we will investigate the effects of urinary phthalate metabolite levels on sexual development and sex hormone levels of 12-year-old children, including gonadal development of both boys and girls, and will measure the major markers of sexual maturation, including AMH and inhibin B.

## Conclusion

*In utero* phthalate exposure did not significantly alter birth outcomes, growth, or reproductive function and development in pre-pubertal children. Furthermore, environmental exposure to certain phthalate metabolites (i.e., MEHP, MnBP, and MBzP) appears to affect reproductive hormone levels in pre-pubertal girls. Our results suggest that future studies focusing on the environmental health effects of phthalates and their metabolites are warranted.

## Supporting Information

Table S1 **Birth outcomes, including birth weights, birth lengths, and head circumferences, of newborns at different gestational ages.**
(DOC)

Table S2 **Z- scores of birth outcomes of newborns at different gestational ages.**
(DOC)

Table S3 **Correlation analysis of maternal urinary levels of phthalate metabolites with birth outcome and the phthalate metabolites levels of eight-year-old children.**
(DOC)

Table S4 **Correlation analysis of maternal urinary levels of phthalate metabolites with physical characteristics and reproductive development of children at 8 years of age.**
(DOC)

Table S5 **Correlation analysis of the urinary phthalate metabolite levels of eight-year-old children with their physical characteristics and reproductive development.** Raw data (Sheet 1) and code book (Sheet 2) in an Excel file “Original data.xls”.
(DOC)
