# Supplementary material for: Sex Steroid Hormone Levels and Reproductive Development of Eight-Year-Old Children following In Utero and Environmental Exposure to Phthalates
Source: PLoS One. 2014 Sep 10;9(9):e102788. doi: 10.1371/journal.pone.0102788 (PMC4160173; doi:10.1371/journal.pone.0102788)
Supplement: Table S1 — Birth outcomes, including birth weights, birth lengths, and head circumferences, of newborns at different gestational ages. (DOC) [file pone.0102788.s001.doc]

**Table S1:** Birth outcomes, including birth weights, birth lengths, and head circumferences, of newborns at different gestational ages.

| Gestational age (weeks) | n | Birth weight (g) | Birth length (cm) | Head circumference (cm) |
| --- | --- | --- | --- | --- |
| 26 | 1 | 3150.00 | 49.00 | 34.0000 |
| 29 | 1 | 1878.00 | 44.00 | 36.0000 |
| 36 | 1 | 2740.00 | 49.50 | 35.5000 |
| 37 | 10 | 2655.00±393.31 | 49.20±2.41 | 32.85±1.00 |
| 38 | 17 | 3147.06±360.34 | 51.94±1.88 | 33.47±1.34 |
| 39 | 26 | 3166.15±323.02 | 51.87±2.42 | 33.65±1.14 |
| 40 | 19 | 3322.11±344.55 | 51.97±2.21 | 33.55±1.18 |
| 41 | 6 | 3335.00±408.60 | 51.75±1.54 | 34.00±0.89 |
| 42 | 2 | 3420.00±608.11 | 52.50±0.71 | 34.00±2.83 |
| Total | 83 | 3133.83±421.31 | 51.43±2.44 | 33.58±1.23 |

Data are presented as mean±SD.
