# Supplementary material for: Sex Steroid Hormone Levels and Reproductive Development of Eight-Year-Old Children following In Utero and Environmental Exposure to Phthalates
Source: PLoS One. 2014 Sep 10;9(9):e102788. doi: 10.1371/journal.pone.0102788 (PMC4160173; doi:10.1371/journal.pone.0102788)
Supplement: Table S2 — Z- scores of birth outcomes of newborns at different gestational ages. (DOC) [file pone.0102788.s002.doc]

**Table S2**: Z- scores of birth outcomes of newborns at different gestational ages.

| Gestational age (weeks) | n | Birth weight (g) | Birth length (cm) | Head circumference (cm) |
| --- | --- | --- | --- | --- |
| ≤37 | 13 | 0.0000±0.9999 | -0.0009±0.9990 | 0.0033±1.0012 |
| 38 | 17 | 0.0000±1.0000 | 0.0005±0.7761 | 0.0004±1.0001 |
| 39 | 26 | 0.0000±1.0000 | -0.0019±0.9978 | 0.0034±.9984 |
| 40 | 19 | 0.0000±1.0000 | 0.0017±1.0018 | 0.0022±0.9977 |
| 41 | 6 | 0.0000±1.0000 | 0.0000±1.0007 | 0.0000±1.0050 |
| 42 | 2 | 0.0000±1.0000 | 0.0000±0.9959 | 0.0000±0.9994 |
| Total | 83 | 0.0000±0.9690 | -0.0003±0.9277 | 0.0022±0.9685 |

Data are presented as mean±SD.
