# Supplementary material for: Sex Steroid Hormone Levels and Reproductive Development of Eight-Year-Old Children following In Utero and Environmental Exposure to Phthalates
Source: PLoS One. 2014 Sep 10;9(9):e102788. doi: 10.1371/journal.pone.0102788 (PMC4160173; doi:10.1371/journal.pone.0102788)
Supplement: Table S3 — Correlation analysis of maternal urinary levels of phthalate metabolites with birth outcome and the phthalate metabolites levels of eight-year-old children. (DOC) [file pone.0102788.s003.doc]

**Table S3:** Correlation analysis of maternal urinary levels of phthalate metabolites with birth outcome and the phthalate metabolites levels of eight-year-old children.

|  |  | **MEHP** | |  | **5OH-MEHP** | |  | **5oxo-MEHP** | |  | **Total DEHP** | |  | **MnBP** | |  | **MBzP** | |  | **MMP** | |  | **MEP** | |
| --- | --- | --- | --- | --- | --- | --- | --- | --- | --- | --- | --- | --- | --- | --- | --- | --- | --- | --- | --- | --- | --- | --- | --- | --- |
| **Population** | **Outcomes** | **r** | ***P* -value** |  | **r** | ***P* -value** |  | **r** | ***P* -value** |  | **r** | ***P* -value** |  | **r** | ***P* -value** |  | **r** | ***P* -value** |  | **r** | ***P* -value** |  | **r** | ***P* -value** |
| Total (n=130) |  |  |  |  |  |  |  |  |  |  |  |  |  |  |  |  |  |  |  |  |  |  |  |  |
|  | ***Birth outcomes*** |  |  |  |  |  |  |  |  |  |  |  |  |  |  |  |  |  |  |  |  |  |  |  |
|  | Gestational age, weeks | 0.142 | 0.193 |  | 0.099 | 0.364 |  | 0.137 | 0.207 |  | 0.163 | 0.134 |  | 0.056 | 0.609 |  | 0.175 | 0.107 |  | 0.004 | 0.974 |  | 0.153 | 0.160 |
|  | Birth weight, g ^a^ | 0.039 | 0.726 |  | 0.157 | 0.155 |  | 0.138 | 0.213 |  | 0.156 | 0.160 |  | 0.179 | 0.105 |  | -0.119 | 0.283 |  | -0.011 | 0.922 |  | 0.029 | 0.794 |
|  | Birth length, cm ^a^ | 0.059 | 0.593 |  | 0.035 | 0.753 |  | 0.030 | 0.791 |  | 0.081 | 0.468 |  | 0.139 | 0.211 |  | -0.087 | 0.436 |  | -0.044 | 0.692 |  | -0.039 | 0.726 |
|  | Head circumference, cm ^a^ | 0.071 | 0.525 |  | 0.101 | 0.364 |  | 0.035 | 0.752 |  | 0.077 | 0.486 |  | 0.069 | 0.535 |  | -0.081 | 0.464 |  | 0.692 | 0.643 |  | 0.094 | 0.400 |
|  | Sex (M vs. F) | 0.065 | 0.526 |  | 0.114 | 0.265 |  | 0.132 | 0.196 |  | 0.112 | 0.275 |  | -0.176 | 0.084 |  | -0.176 | 0.084 |  | 0.069 | 0.502 |  | -0.009 | 0.932 |
|  | Phthalate metabolite levels at 8 years of age | 0.019 | 0.829 |  | 0.064 | 0.468 |  | 0.073 | 0.410 |  | 0.046 | 0.604 |  | 0.190 | 0.030 |  | 0.068 | 0.130 |  | 0.020 | 0.821 |  | -0.076 | 0.387 |
| Boys (n=61) |  |  |  |  |  |  |  |  |  |  |  |  |  |  |  |  |  |  |  |  |  |  |  |  |
|  | ***Birth outcomes*** |  |  |  |  |  |  |  |  |  |  |  |  |  |  |  |  |  |  |  |  |  |  |  |
|  | Gestational age, weeks | -0.008 | 0.960 |  | 0.338 | 0.025 |  | 0.307 | 0.043 |  | 0.211 | 0.169 |  | 0.013 | 0.931 |  | 0.270 | 0.076 |  | -0.044 | 0.776 |  | 0.94 | 0.940 |
|  | Birth weight, g ^a^ | 0.079 | 0.625 |  | 0.225 | 0.157 |  | 0.251 | 0.113 |  | 0.330 | 0.035 |  | 0.236 | 0.137 |  | -0.208 | 0.192 |  | **-0.423** | **0.006*** |  | 0.038 | 0.816 |
|  | Birth length, cm ^a^ | 0.129 | 0.420 |  | 0.197 | 0.217 |  | 0.214 | 0.179 |  | 0.322 | 0.040 |  | 0.385 | 0.013 |  | -0.083 | 0.605 |  | -0.111 | 0.491 |  | 0.067 | 0.677 |
|  | Head circumference, cm ^a^ | 0.127 | 0.427 |  | 0.381 | 0.014 |  | 0.312 | 0.047 |  | 0.360 | 0.021 |  | 0.199 | 0.213 |  | -0.061 | 0.703 |  | -0.393 | 0.011 |  | 0.192 | 0.228 |
|  | Phthalate metabolite levels at 8 years of age | -0.118 | 0.366 |  | 0.185 | 0.154 |  | 0.751 | 0.751 |  | 0.028 | 0.829 |  | 0.089 | 0.496 |  | 0.138 | 0.290 |  | -0.130 | 0.319 |  | -0.087 | 0.507 |
| Girls (n=69) |  |  |  |  |  |  |  |  |  |  |  |  |  |  |  |  |  |  |  |  |  |  |  |  |
|  | ***Birth outcomes*** |  |  |  |  |  |  |  |  |  |  |  |  |  |  |  |  |  |  |  |  |  |  |  |
|  | Gestational age, weeks | 0.249 | 0.112 |  | -0.163 | 0.303 |  | -0.027 | 0.864 |  | 0.073 | 0.645 |  | 0.056 | 0.724 |  | 0.080 | 0.615 |  | 0.051 | 0.750 |  | 0.323 | 0.037 |
|  | Birth weight, g ^a^ | 0.025 | 0.874 |  | 0.004 | 0.982 |  | -0.036 | 0.819 |  | -0.038 | 0.812 |  | 0.167 | 0.290 |  | 0.037 | 0.817 |  | 0.320 | 0.039 |  | 0.073 | 0.648 |
|  | Birth length, cm ^a^ | 0.027 | 0.868 |  | -0.147 | 0.352 |  | -0.167 | 0.290 |  | -0.141 | 0.372 |  | -0.029 | 0.856 |  | -0.039 | 0.804 |  | 0.004 | 0.979 |  | -0.096 | 0.546 |
|  | Head circumference, cm ^a^ | 0.051 | 0.749 |  | -0.152 | 0.338 |  | -0.202 | 0.200 |  | -0.155 | 0.326 |  | 0 | 0.999 |  | -0.036 | 0.821 |  | 0.132 | 0.403 |  | 0.024 | 0.878 |
|  | Phthalate metabolite levels at 8 years of age | 0.089 | 0.469 |  | -0.062 | 0.615 |  | 0.096 | 0.430 |  | 0.075 | 0.542 |  | 0.291 | 0.015 |  | 0.038 | 0.758 |  | 0.155 | 0.203 |  | -0.102 | 0.405 |

Data were analyzed using the Spearman’s correlation analysis, with the coefficient of correlation (r) and corresponding p-value for each correlation presented.

^a^ Z-scores of birth outcomes, including body weight, body length, and head circumference, for gestational age were calculated prior to conducting correlation analysis.

* *P*<0.00625 (0.05/8) indicates a significant correlation.

*Abbreviations:* MEHP, mono-2-ethylhexyl phthalate; 5OH-MEHP, mono-(2-ethyl-5-hydroxyhexyl) phthalate; 5oxo-MEHP, mono-(2-ethyl-5-oxohexyl) phthalate; DEHP, di-(2-ethylhexyl) phthalate; MnBP, mono-n-butyl phthalate; MBzP, mono-benzyl phthalate; MMP, monomethyl phthalate; MEP, mono-ethyl phthalate.
