# Supplementary material for: Sex Steroid Hormone Levels and Reproductive Development of Eight-Year-Old Children following In Utero and Environmental Exposure to Phthalates
Source: PLoS One. 2014 Sep 10;9(9):e102788. doi: 10.1371/journal.pone.0102788 (PMC4160173; doi:10.1371/journal.pone.0102788)
Supplement: Table S4 — Correlation analysis of maternal urinary levels of phthalate metabolites with physical characteristics and reproductive development of children at 8 years of age. (DOC) [file pone.0102788.s004.doc]

**Table S4:** Correlation analysis of maternal urinary levels of phthalate metabolites with physical characteristics and reproductive development of children at 8 years of age.

|  |  | **MEHP** | |  | **5OH-MEHP** | |  | **5oxo-MEHP** | |  | **Total DEHP** | |  | **MnBP** | |  | **MBzP** | |  | **MMP** | |  | **MEP** | |
| --- | --- | --- | --- | --- | --- | --- | --- | --- | --- | --- | --- | --- | --- | --- | --- | --- | --- | --- | --- | --- | --- | --- | --- | --- |
| **Population** | **Outcomes** | **r** | ***P* -value** |  | **r** | ***P* -value** |  | **r** | ***P* -value** |  | **r** | ***P* -value** |  | **r** | ***P* -value** |  | **r** | ***P* -value** |  | **r** | ***P* -value** |  | **r** | ***P* -value** |
| Total (n=130) |  |  |  |  |  |  |  |  |  |  |  |  |  |  |  |  |  |  |  |  |  |  |  |  |
|  | Bone age | -0.027 | 0.763 |  | -0.049 | 0.577 |  | -0.006 | 0.941 |  | -0.006 | 0.947 |  | -0.024 | 0.787 |  | -0.044 | 0.622 |  | -0.112 | 0.206 |  | -0.073 | 0.408 |
|  | Bone age/chronological age ratio | -0.023 | 0.791 |  | -0.044 | 0.623 |  | 0.002 | 0.981 |  | 0.002 | 0.983 |  | -0.012 | 0.894 |  | -0.027 | 0.762 |  | -0.101 | 0.253 |  | -0.07 | 0.430 |
|  | Estradiol, ng/dL | 0.062 | 0.493 |  | 0.053 | 0.553 |  | -0.001 | 0.989 |  | 0.003 | 0.969 |  | 0.008 | 0.926 |  | 0.129 | 0.150 |  | 0.046 | 0.608 |  | 0.072 | 0.421 |
|  | Follicle-stimulating hormone, mIU/mL | -0.093 | 0.303 |  | -0.079 | 0.382 |  | -0.043 | 0.635 |  | -0.102 | 0.259 |  | -0.046 | 0.614 |  | -0.135 | 0.133 |  | -0.032 | 0.720 |  | -0.149 | 0.098 |
|  | Testosterone, pg/mL | -0.138 | 0.125 |  | 0.035 | 0.701 |  | 0.036 | 0.691 |  | -0.023 | 0.800 |  | -0.040 | 0.657 |  | 0.019 | 0.833 |  | 0.034 | 0.705 |  | -0.041 | 0.649 |
|  | Progesterone, ng/mL | -0.019 | 0.830 |  | 0.038 | 0.676 |  | -0.065 | 0.472 |  | -0.029 | 0.746 |  | -0.036 | 0.688 |  | 0.126 | 0.160 |  | 0.011 | 0.902 |  | -0.026 | 0.773 |
|  | Free total testosterone, ng/mL | -0.202 | 0.025 |  | -0.148 | 0.103 |  | -0.142 | 0.118 |  | -0.193 | 0.032 |  | -0.082 | 0.366 |  | -0.149 | 0.100 |  | -0.020 | 0.827 |  | -0.103 | 0.259 |
| Boys (n=61) |  |  |  |  |  |  |  |  |  |  |  |  |  |  |  |  |  |  |  |  |  |  |  |  |
|  | Bone age | -0.03 | 0.819 |  | 0.150 | 0.250 |  | 0.163 | 0.209 |  | 0.155 | 0.233 |  | 0.127 | 0.330 |  | -0.153 | 0.239 |  | -0.285 | 0.026 |  | -0.068 | 0.601 |
|  | Bone age/chronological age ratio | -0.025 | 0.848 |  | 0.149 | 0.253 |  | 0.161 | 0.216 |  | 0.153 | 0.238 |  | 0.128 | 0.325 |  | -0.151 | 0.246 |  | -0.281 | 0.028 |  | -0.065 | 0.618 |
|  | Estradiol, ng/dL | 0.128 | 0.332 |  | 0.214 | 0.101 |  | 0.057 | 0.664 |  | 0.094 | 0.476 |  | 0.144 | 0.271 |  | 0.186 | 0.155 |  | 0.095 | 0.472 |  | 0.277 | 0.032 |
|  | Follicle-stimulating hormone, mIU/mL | -0.01 | 0.941 |  | -0.007 | 0.960 |  | 0.032 | 0.806 |  | 0.068 | 0.605 |  | -0.027 | 0.835 |  | -0.049 | 0.711 |  | -0.070 | 0.598 |  | -0.058 | 0.662 |
|  | Testosterone, pg/mL | -0.188 | 0.150 |  | 0.077 | 0.559 |  | 0.101 | 0.444 |  | 0.001 | 0.994 |  | 0.020 | 0.880 |  | -0.176 | 0.178 |  | 0.203 | 0.119 |  | 0.117 | 0.374 |
|  | Progesterone, ng/mL | 0.104 | 0.428 |  | 0.337 | 0.009 |  | 0.137 | 0.296 |  | 0.174 | 0.185 |  | 0.227 | 0.081 |  | 0.172 | 0.188 |  | 0.165 | 0.207 |  | 0.16 | 0.221 |
|  | Free total testosterone, ng/mL | -0.079 | 0.557 |  | -0.189 | 0.155 |  | -0.109 | 0.417 |  | -0.188 | 0.157 |  | -0.044 | 0.745 |  | -0.110 | 0.410 |  | -0.008 | 0.955 |  | 0.014 | 0.916 |
| Girls (n=69) |  |  |  |  |  |  |  |  |  |  |  |  |  |  |  |  |  |  |  |  |  |  |  |  |
|  | Bone age | -0.098 | 0.422 |  | -0.047 | 0.700 |  | -0.020 | 0.871 |  | -0.044 | 0.718 |  | -0.150 | 0.219 |  | -0.137 | 0.260 |  | -0.050 | 0.681 |  | -0.245 | 0.043 |
|  | Bone age/chronological age ratio | -0.093 | 0.448 |  | -0.043 | 0.725 |  | -0.009 | 0.942 |  | -0.032 | 0.795 |  | -0.140 | 0.250 |  | -0.119 | 0.329 |  | -0.050 | 0.681 |  | -0.248 | 0.040 |
|  | Estradiol, ng/dL | -0.004 | 0.973 |  | -0.084 | 0.500 |  | -0.049 | 0.695 |  | -0.071 | 0.569 |  | -0.071 | 0.571 |  | 0.083 | 0.510 |  | 0.027 | 0.831 |  | -0.121 | 0.333 |
|  | Follicle-stimulating hormone, mIU/mL | -0.114 | 0.370 |  | -0.118 | 0.351 |  | -0.091 | 0.470 |  | -0.195 | 0.120 |  | -0.030 | 0.813 |  | -0.168 | 0.180 |  | -0.002 | 0.989 |  | -0.21 | 0.092 |
|  | Testosterone, pg/mL | -0.104 | 0.404 |  | 0.030 | 0.808 |  | 0.003 | 0.978 |  | -0.045 | 0.720 |  | -0.080 | 0.525 |  | 0.079 | 0.527 |  | -0.037 | 0.770 |  | -0.145 | 0.244 |
|  | Progesterone, ng/mL | -0.115 | 0.360 |  | -0.209 | 0.092 |  | -0.261 | 0.034* |  | -0.222 | 0.073 |  | -0.183 | 0.142 |  | 0.068 | 0.586 |  | -0.080 | 0.524 |  | -0.188 | 0.130 |
|  | Free total testosterone, ng/mL | -0.289 | 0.020 |  | -0.098 | 0.438 |  | -0.176 | 0.160 |  | -0.204 | 0.103 |  | -0.081 | 0.521 |  | -0.156 | 0.214 |  | -0.006 | 0.961 |  | -0.204 | 0.103 |
|  | Tanner stage (1-3) | -0.163 | 0.182 |  | 0.102 | 0.404 |  | 0.074 | 0.544 |  | -0.009 | 0.939 |  | -0.075 | 0.540 |  | -0.175 | 0.149 |  | -0.147 | 0.228 |  | -0.049 | 0.689 |

Data were analyzed using the Spearman’s correlation analysis, with the coefficient of correlation (r) and corresponding p-value for each correlation presented.

^a^ Z-scores of birth outcomes, including body weight, body length, and head circumference, for gestational age were calculated prior to conducting correlation analysis.

* *P*<0.00625 (0.05/8) indicates a significant correlation.

*Abbreviations:* MEHP, mono-2-ethylhexyl phthalate; 5OH-MEHP, mono-(2-ethyl-5-hydroxyhexyl) phthalate; 5oxo-MEHP, mono-(2-ethyl-5-oxohexyl) phthalate; DEHP, di-(2-ethylhexyl) phthalate; MnBP, mono-n-butyl phthalate; MBzP, mono-benzyl phthalate; MMP, monomethyl phthalate; MEP, mono-ethyl phthalate.
