# Supplementary material for: Sex Steroid Hormone Levels and Reproductive Development of Eight-Year-Old Children following In Utero and Environmental Exposure to Phthalates
Source: PLoS One. 2014 Sep 10;9(9):e102788. doi: 10.1371/journal.pone.0102788 (PMC4160173; doi:10.1371/journal.pone.0102788)
Supplement: Table S5 — Correlation analysis of the urinary phthalate metabolite levels of eight-year-old children with their physical characteristics and reproductive development. Raw data (Sheet 1) and code book (Sheet 2) in an Excel file “Original data.xls”. (DOC) [file pone.0102788.s005.doc]

**Table S5:** Correlation analysis of the urinary phthalate metabolite levels of eight-year-old children with their physical characteristics and reproductive development.

|  |  | **MEHP** | |  | **5OH-MEHP** | |  | **5oxo-MEHP** | |  | **Total DEHP** | |  | **MnBP** | |  | **MBzP** | |  | **MMP** | |  | **MEP** | |
| --- | --- | --- | --- | --- | --- | --- | --- | --- | --- | --- | --- | --- | --- | --- | --- | --- | --- | --- | --- | --- | --- | --- | --- | --- |
| **Population** | **Outcomes** | **r** | ***P* -value** |  | **r** | ***P* -value** |  | **r** | ***P* -value** |  | **r** | ***P* -value** |  | **r** | ***P* -value** |  | **r** | ***P* -value** |  | **r** | ***P* -value** |  | **r** | ***P* -value** |
| Total (n=130) |  |  |  |  |  |  |  |  |  |  |  |  |  |  |  |  |  |  |  |  |  |  |  |  |
|  | Bone age | 0.095 | 0.281 |  | -0.021 | 0.813 |  | -0.102 | 0.249 |  | -0.064 | 0.473 |  | 0.010 | 0.906 |  | -0.113 | 0.202 |  | 0.079 | 0.372 |  | 0.095 | 0.281 |
|  | Bone age/chronological age ratio | 0.097 | 0.271 |  | -0.025 | 0.779 |  | -0.100 | 0.257 |  | -0.065 | 0.464 |  | 0.019 | 0.830 |  | -0.118 | 0.182 |  | 0.078 | 0.378 |  | 0.097 | 0.271 |
|  | Estradiol, ng/dL | 0.047 | 0.604 |  | 0.005 | 0.955 |  | 0.052 | 0.565 |  | 0.008 | 0.926 |  | 0.009 | 0.922 |  | -0.023 | 0.799 |  | -0.101 | 0.263 |  | 0.047 | 0.604 |
|  | Follicle-stimulating hormone, mIU/mL | -0.161 | 0.070 |  | -0.114 | 0.204 |  | -0.084 | 0.354 |  | -0.085 | 0.343 |  | 0.037 | 0.684 |  | 0.065 | 0.474 |  | 0.075 | 0.405 |  | -0.161 | 0.073 |
|  | Testosterone, pg/mL | -0.046 | 0.606 |  | -0.046 | 0.609 |  | -0.018 | 0.840 |  | -0.032 | 0.722 |  | -0.034 | 0.705 |  | 0.071 | 0.428 |  | 0.020 | 0.826 |  | -0.046 | 0.606 |
|  | Progesterone, ng/mL | 0.108 | 0.227 |  | -0.060 | 0.508 |  | -0.009 | 0.917 |  | -0.048 | 0.595 |  | 0.036 | 0.688 |  | -0.038 | 0.676 |  | -0.003 | 0.969 |  | 0.108 | 0.227 |
|  | Free total testosterone, ng/mL | 0.100 | 0.272 |  | -0.042 | 0.644 |  | -0.106 | 0.244 |  | -0.014 | 0.875 |  | 0.004 | 0.967 |  | 0.010 | 0.910 |  | 0.056 | 0.540 |  | 0.1 | 0.272 |
| Boys (n=61) |  |  |  |  |  |  |  |  |  |  |  |  |  |  |  |  |  |  |  |  |  |  |  |  |
|  | Bone age | 0.072 | 0.579 |  | 0.085 | 0.513 |  | 0.067 | 0.610 |  | 0.062 | 0.635 |  | 0.056 | 0.667 |  | 0.208 | 0.107 |  | 0.240 | 0.062 |  | 0.072 | 0.579 |
|  | Bone age/chronological age ratio | 0.074 | 0.570 |  | 0.088 | 0.502 |  | 0.069 | 0.595 |  | 0.065 | 0.618 |  | 0.054 | 0.678 |  | 0.210 | 0.105 |  | 0.243 | 0.059 |  | 0.074 | 0.570 |
|  | Estradiol, ng/dL | -0.054 | 0.681 |  | 0.039 | 0.770 |  | 0.094 | 0.476 |  | 0.028 | 0.834 |  | -0.131 | 0.318 |  | -0.204 | 0.119 |  | -0.108 | 0.411 |  | -0.054 | 0.681 |
|  | Follicle-stimulating hormone, mIU/mL | -0.247 | 0.058 |  | -0.179 | 0.171 |  | -0.183 | 0.162 |  | -0.167 | 0.202 |  | -0.107 | 0.417 |  | -0.134 | 0.308 |  | -0.066 | 0.619 |  | -0.247 | 0.058 |
|  | Testosterone, pg/mL | -0.156 | 0.235 |  | -0.069 | 0.599 |  | -0.027 | 0.840 |  | -0.034 | 0.795 |  | -0.080 | 0.545 |  | 0.123 | 0.350 |  | 0.056 | 0.669 |  | -0.156 | 0.235 |
|  | Progesterone, ng/mL | 0.042 | 0.752 |  | -0.018 | 0.894 |  | -0.030 | 0.819 |  | -0.059 | 0.652 |  | -0.117 | 0.372 |  | -0.147 | 0.264 |  | -0.098 | 0.454 |  | 0.042 | 0.752 |
|  | Free total testosterone, ng/mL | 0.207 | 0.119 |  | -0.103 | 0.444 |  | -0.146 | 0.275 |  | -0.06 | 0.655 |  | 0.055 | 0.680 |  | 0.080 | 0.549 |  | 0.113 | 0.397 |  | 0.207 | 0.119 |
| Girls (n=69) |  |  |  |  |  |  |  |  |  |  |  |  |  |  |  |  |  |  |  |  |  |  |  |  |
|  | Bone age | -0.111 | 0.366 |  | -0.042 | 0.735 |  | -0.179 | 0.140 |  | -0.093 | 0.450 |  | -0.187 | 0.125 |  | -0.316 | 0.008* |  | -0.135 | 0.269 |  | -0.111 | 0.366 |
|  | Bone age/chronological age ratio | -0.113 | 0.357 |  | -0.043 | 0.727 |  | -0.167 | 171.000 |  | -0.089 | 0.467 |  | -0.172 | 0.156 |  | -0.305 | 0.011 |  | -0.146 | 0.233 |  | -0.113 | 0.357 |
|  | Estradiol, ng/dL | 0.071 | 0.570 |  | -0.060 | 0.633 |  | 0.018 | 0.885 |  | -0.011 | 0.931 |  | 0.090 | 0.475 |  | 0.156 | 0.210 |  | -0.137 | 0.273 |  | 0.071 | 0.570 |
|  | Follicle-stimulating hormone, mIU/mL | -0.085 | 0.501 |  | -0.069 | 0.587 |  | -0.014 | 0.913 |  | -0.033 | 0.791 |  | 0.151 | 0.230 |  | 0.246 | 0.048 |  | 0.214 | 0.087 |  | -0.085 | 0.501 |
|  | Testosterone, pg/mL | -0.022 | 0.860 |  | -0.032 | 0.799 |  | -0.002 | 0.986 |  | -0.022 | 0.864 |  | -0.047 | 0.710 |  | 0.030 | 0.808 |  | -0.037 | 0.767 |  | -0.022 | 0.860 |
|  | Progesterone, ng/mL | 0.138 | 0.269 |  | -0.082 | 0.512 |  | 0.037 | 0.768 |  | -0.017 | 0.892 |  | 0.162 | 0.195 |  | 0.052 | 0.679 |  | 0.058 | 0.642 |  | 0.138 | 0.269 |
|  | Free total testosterone, ng/mL | 0.008 | 0.950 |  | 0.032 | 0.803 |  | -0.096 | 0.445 |  | 0.001 | 0.996 |  | -0.038 | 0.766 |  | -0.060 | 0.633 |  | 0.022 | 0.862 |  | 0.008 | 0.950 |
|  | Tanner stage (1-3) | 0.136 | 0.266 |  | 0.031 | 0.800 |  | 0.004 | 0.973 |  | 0.013 | 0.913 |  | -0.097 | 0.427 |  | -0.080 | 0.515 |  | -0.057 | 0.642 |  | 0.136 | 0.266 |

Data were analyzed using the Spearman’s correlation analysis, with the coefficient of correlation (r) and corresponding p-value for each correlation presented.

^a^ Z-scores of birth outcomes, including body weight, body length, and head circumference, for gestational age were calculated prior to conducting correlation analysis.

* *P*<0.00625 (0.05/8) indicates a significant correlation.

*Abbreviations:* MEHP, mono-2-ethylhexyl phthalate; 5OH-MEHP, mono-(2-ethyl-5-hydroxyhexyl) phthalate; 5oxo-MEHP, mono-(2-ethyl-5-oxohexyl) phthalate; DEHP, di-(2-ethylhexyl) phthalate; MnBP, mono-n-butyl phthalate; MBzP, mono-benzyl phthalate; MMP, monomethyl phthalate; MEP, mono-ethyl phthalate.
